# Unraveling microbial processes involved in carbon and nitrogen cycling and greenhouse gas emissions in rewetted peatlands by molecular biology

**DOI:** 10.1007/s10533-024-01122-6

**Published:** 2024-03-16

**Authors:** Emilie Gios, Erik Verbruggen, Joachim Audet, Rachel Burns, Klaus Butterbach-Bahl, Mikk Espenberg, Christian Fritz, Stephan Glatzel, Gerald Jurasinski, Tuula Larmola, Ülo Mander, Claudia Nielsen, Andres F. Rodriguez, Clemens Scheer, Dominik Zak, Hanna M. Silvennoinen

**Affiliations:** 1 NINA, Norwegian Institute for Nature Research, PO Box 5685, Torgarden, NO-7485 Trondheim, Norway; 2 Plants and Ecosystems Research Group, Department of Biology, University of Antwerp, Universiteitsplein 1, Wilrijk, 2610 Antwerp, Belgium; 3 Department of Ecoscience, Aarhus University, C.F. Møllers Allé, 8000 Aarhus, Denmark; 4 Department of Geosciences and Natural Resource Management, University of Copenhagen, 1350 Copenhagen, Denmark; 5 Institute of Meteorology and Climate Research, Atmospheric Environmental Research, Karlsruhe Institute of Technology, 82467 Garmisch-Partenkirchen, Germany; 6 Department of Agroecology, Pioneer Center for Research in Sustainable Agricultural Futures (Land-CRAFT), Aarhus University, 8000 Aarhus, Denmark; 7 Department of Geography, Institute of Ecology and Earth Sciences, University of Tartu, 46 St., Vanemuise, 51003 Tartu, Estonia; 8 Aquatic Ecology and Environmental Biology, Radboud Institute for Biological and Environmental Sciences (RIBES), Radboud University, Heyendaalseweg 135, 6525 AJ Nijmegen, The Netherlands; 9 Department of Geography and Regional Research, University of Vienna, Althanstraße 14, 1090 Vienna, Austria; 10 Faculty of Agriculture and Environment, Landscape Ecology and Site Evaluation, University of Rostock, Justus-von-Liebig-Weg 6, 18059 Rostock, Germany; 11 Department of Maritime Systems, Faculty of Interdisciplinary Research, University of Rostock, Albert- Einstein-Straße 3, 18059 Rostock, Germany; 12 Natural Resources Institute Finland (Luke), 00790 Helsinki, Finland; 13 Department of Agroecology, Faculty of Technical Sciences, Aarhus University, Blichers Alle 20, 8830 Tjele, Denmark; 14 CBIO, Centre for Circular Bioeconomy, Aarhus University, 8830 Tjele, Denmark; 15 Department of Ecohydrology and Biogeochemistry, Leibniz-Institute of Freshwater Ecology and Inland Fisheries, Müggelseedamm 301, 12587 Berlin, Germany

**Keywords:** Peatland rewetting, Microbial communities, Biogeochemical processes, Molecular biology, Climate change mitigation

## Abstract

Restoration of drained peatlands through rewetting has recently emerged as a prevailing strategy to mitigate excessive greenhouse gas emissions and re-establish the vital carbon sequestration capacity of peatlands. Rewetting can help to restore vegetation communities and biodiversity, while still allowing for extensive agricultural management such as paludiculture. Belowground processes governing carbon fluxes and greenhouse gas dynamics are mediated by a complex network of microbial communities and processes. Our understanding of this complexity and its multi-factorial controls in rewetted peatlands is limited. Here, we summarize the research regarding the role of soil microbial communities and functions in driving carbon and nutrient cycling in rewetted peatlands including the use of molecular biology techniques in understanding biogeochemical processes linked to greenhouse gas fluxes. We emphasize that rapidly advancing molecular biology approaches, such as high-throughput sequencing, are powerful tools helping to elucidate the dynamics of key biogeochemical processes when combined with isotope tracing and greenhouse gas measuring techniques. Insights gained from the gathered studies can help inform efficient monitoring practices for rewetted peatlands and the development of climate-smart restoration and management strategies.

**Supplementary Information:**

The online version contains supplementary material available at 10.1007/s10533-024-01122-6.

## Introduction

Approximately 12% of the world’s peatlands have been subject to drainage for the purpose of agriculture, peat extraction, urbanization, or forestry (UNEP 2022). Peatland drainage causes land subsidence, peat loss, greenhouse gas (GHG) emissions and eutrophication of water bodies, leading to a loss of almost all ecological functions (Kreyling et al. 2021). The destruction and subsequent loss of peatland ecosystems and the benefits they provide continues at a rate greater than for any other ecosystem type, including tropical rainforests (Loisel et al. 2021). Through aerobic peat mineralization and increased incidence of fires, drained peatlands emit about 1.9 Gt carbon dioxide (CO_2_)-equivalents per year globally (Leifeld and Menichetti 2018), corresponding to 10% of the GHG emissions from agriculture, land-use change and forestry combined (IPCC 2014).

In the last decades, the understanding of biogeochemical processes in rewetted degraded peatlands has increased substantially with new insights gained from monitoring programs accompanying peatland restoration projects. This information has allowed to outline the environmental implications of different restoration measures and to provide guidelines to optimize restoration (Jurasinski et al. 2020; Kreyling et al. 2021). Key driving factors are water table position and source of water (e.g., rainfall, groundwater), while chemical composition of discharging water, physico-chemical soil characteristics, dominant vegetation type and eventually composition of the microbial community are important indicators of ongoing biogeochemical processes (Wen et al. 2018; Walton et al. 2020; Evans et al. 2021). Depending on drainage history and specific characteristics of sites under consideration, such as size, landscape position, soil properties, land use, and the presence of valuable species, different rewetting strategies might be appropriate. For example, topsoil removal or gradual rising of water table in combination with a new form of wetland use called paludiculture (Zak et al. 2018) can be considered. There is no “one-size fits it all” restoration solution; hence, each approach has its own merits and applications (Zak and Mc Innes 2022).

Molecular biology techniques are powerful tools allowing to directly target microbial processes of interest and, thus, contribute to fostering a comprehensive understanding of biodiversity and ecosystem functioning. Gene-targeted approaches can be used to identify taxonomic biodiversity of microorganisms (sequencing barcode regions on e.g., the 16S rRNA and 18S rRNA genes), or to explore the prevalence and changes in specific functions (targeting genes coding for enzymes related to biogeochemical cycling for example using quantitative polymerase chain reaction [qPCR]). Metagenomics allows for the analysis of all the genetic material (DNA) present in a sample, providing a comprehensive view of the taxonomic and functional diversity. Transcriptomics provides information about genes that are actively being expressed by microorganisms (by targeting RNA molecules) and avoids inclusion of inactive or even dead genetic material, and enables querying real-time coupling of microbial activity and functional properties.

Here, we provide a comprehensive review of molecular biology methods used to assess microbial functions linked to biodiversity and biogeochemistry in rewetted peatlands. Climate, geology, and legacies from the original pristine state, as well as those from drainage and land use following the drainage form the microbiome of rewetted peatlands. Selection of the restoration method (rewetting/revegetation/paludiculture, see below) will further shape the microbial communities and their functions; this review aims to gather findings from research into how microbial communities respond to this environmental change. We will first summarize the current knowledge on general peat properties affecting the microbiome and its biogeochemical functions in pristine, drained and restored peatlands. We then align the recent literature on GHG emissions and biogeochemical processes, and how these findings link to microbial functions as explored by molecular biology methods. We, thus, provide a tool that helps general readership to get insights in the current stage of knowledge of microbial biogeochemical linkages with peatland rewetting, and ultimately help planning future studies in this field.

## Effects of drainage and rewetting on peat biogeochemistry

Peat types and their characteristics are largely controlled by natural hydrology in pristine peatlands. Rainwater-fed bogs (ombrotrophic peatlands) are predominantly lower in nutrients and terminal electron acceptors (TEA) compared to groundwater-fed fens. Compared to drained and rewetted peatlands, both bogs and fens are characterized by lower bulk densities, higher carbon (C) contents per dry weight and lower decomposition status of C compounds (amongst other parameters, listed in Table S1 with references).

Predominant land use and intensity of use of peatlands vary between geographical regions, and are controlled by climate, culture, as well as general regional socio-economic conditions. While north-west European peatlands are often heavily drained and used for intensive dairy production (de Vos et al. 2010), those in Northern Europe (Sweden, Finland, Estonia, Latvia, Lithuania) are commonly drained to a shallower extent for forestry (UNEP 2022). Mountainous peatlands on the other side are frequently used in traditional ways such as extensive meadows for pasture (e.g., Sjögren 2006; Jenkins and Walker 2022). Abandoned peat extraction areas can be used for agriculture and berry cultivation (Albert et al. 2011), afforestation (Caisse et al. 2008), and bioenergy production, where the last has been shown as an option to mitigate the atmospheric impact in peatland-rich Northern Europe (Hyvönen et al. 2009; Mander et al. 2012; Espenberg et al. 2016). Drainage causes severe peat degradation and thus impairs the ecological functioning of both bogs and fens (Holden et al. 2004). These drastic hydrological changes lead to aeration of the drained peat layers and thus foster the mineralization of organic matter built up within the last several hundred years, therewith turning peatlands into significant sources of C and nutrients. The non-reversible changes of peat soil characteristics following drainage and the consequences for ecosystem functioning are well investigated (see Table S1).

Restoration of degraded peatlands can be carried out with multiple strategies. Rewetting or “blocking ditches” are the most common measures. Degraded peatlands can in addition be revegetated with vegetation typical to local peatlands and the most decomposed, hydrophobic topsoil may be removed to improve the restoration success. When boreal forestry drained peatlands are restored, in addition to rewetting, depending on the pre-drainage tree cover, the tree layer is partially or completely removed to avoid decaying felling residues hampering the peatland’s recovery toward its nutrient-poor, pristine conditions and to avoid risk of nutrient leaching (Tolvanen et al. 2020). Peatlands can be either rewetted for nature conservation or paludiculture, where, in the latter, plant biomass is harvested for food, feed, fodder or energy after rewetting (Wichtmann and Schäfer 2007). Different restoration strategies most certainly lead to different implications to the microbiome and biogeochemical processes, but few studies exist comparing them. Hereinafter, we refer to restored peatlands as a general term where other restoration strategies than rewetting were used (e.g., rewilding, paludiculture, topsoil removal). Rewetting drained organic soils has been proven to be an effective measure to strongly reduce agricultural CO_2_ emissions and to revert soil carbon sequestration in the short-term (Cabezas et al. 2014; Nugent et al. 2018). Likewise, the nitrogen sink function can be recovered in the short-term as anaerobic conditions are re-established after only a few days of rewetting (Zak and Gelbrecht 2007; Cabezas et al. 2013). On the other hand, there is evidence that rewetted peatlands become strong emitters of methane (CH_4_) (Hahn et al. 2015; Antonievic et al. 2023), nutrients and dissolved organic matter-possibly for decades (Zak and McInnes 2022). Unlike in pristine peatlands, the decomposition of organic matter in rewetted fen peatlands is strongly controlled by the availability of electron acceptors like ferric iron and sulfate, enhanced nutrient availability, circumneutral pH, and lack of polyphenolic substances (Zak et al. 2019). Respiration measurements with different organic substrates from rewetted peatlands suggest that degraded peat without any fresh plant-derived material is relatively inert in terms of decomposition, whereas significant anaerobic production of CO_2_ and CH_4_ in peat may occur only when enough labile organic matter is available either from root turnover or exudation (Hahn-Schöfl et al. 2011). Elevated nutrient levels in degraded peat soil favor the establishment of fast-growing reed communities and, in case of inundated conditions, the formation of highly active detritus mud layers that function as biogeochemical hotspots for nutrient and CH_4_ release (Zak et al. 2018). Overall, revegetation, paludiculture crops and top soil recycling/removal have been shown to be effective measures to reduce the CH_4_ emission potential in (re)flooded peatlands (Huth et al. 2020; Boonman et al. 2023; Quadra et al. 2023).

## Belowground microbial processes involved in GHG dynamics

### Carbon dioxide

**Fig. 1:**
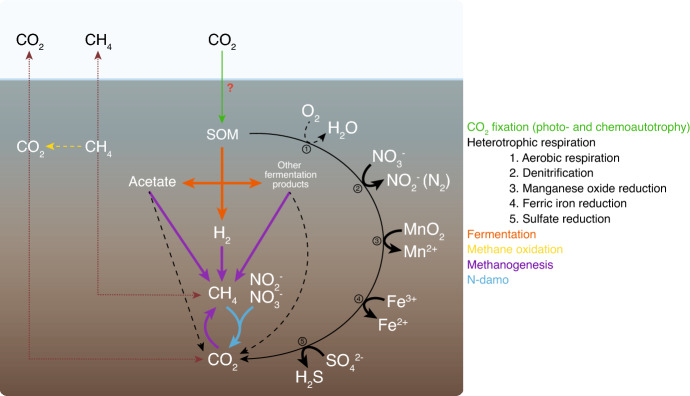
Soil processes involved in carbon dioxide (CO_2_) and methane (CH_4_) fluxes and expected effects of peatland rewetting. Dashed lines correspond to pathways expected to decrease their contribution to GHG fluxes after rewetting and thick lines to increase their contribution. Red dotted lines represent gas diffusion pathways in soil for CO_2_ and CH_4_. Question mark highlights uncertainties regarding the expected effect of rewetting due to lack of information. *N-damo* nitrite-dependent anaerobic methane oxidation, *SOM* soil organic matter

Rewetting of drained organic soils is an effective measure to strongly reduce agricultural CO_2_ emissions and to revert soil C sequestration in the short-term (Cabezas et al. 2014; Nugent et al. 2018). A recent meta-analysis by Darusman et al. (2023) showed that rewetting reduced CO_2_ emissions by 1.43 ± 0.35 Mg CO_2_-C ha^−1^ yr^−1^ on average, but the effects varied depending on climatic zone and nutrient status. Currently, CO_2_ emission factors for rewetted peatlands are between − 0.34 and − 0.55 Mg CO_2_-C ha^−1^ yr^−1^ for boreal peatlands, − 0.23 and 0.5 Mg CO_2_-C ha^−1^ yr^−1^ for temperate peatlands, and 0 Mg CO_2_-C ha^−1^ yr^−1^ for tropical peatlands (Wilson et al. 2016). High nutrient concentrations in peat soils, particularly in temperate peatlands, generate larger CO_2_ emissions from rewetted sites (Wilson et al. 2016; Hemes et al. 2019; Tiemeyer et al. 2020). Additionally, vegetation can affect emissions by adding fresh plant residues to the soil (Rigney et al. 2018) or by transporting O_2_ to the peat through roots (Zhong et al. 2020; Darusman et al. 2023) thereby increasing peat decomposition and CO_2_ emissions. These emissions are governed by the dynamics between CO_2_ uptake by ecosystems, i.e., photosynthesis by plants and soil autotrophic microorganisms (both photo- and chemoautotrophic), and loss to the atmosphere from ecosystem respiration (Fig. 1). The latter includes both autotrophic respiration from plants and microbial heterotrophic respiration.

While this review largely concentrates knowledge on soil microbial processes, which contribute to the breakdown of soil organic matter and resulting CO_2_ emissions, it is important to note that autotrophic respiration by plants constitutes a major component of CO_2_ emissions from the ecosystem to the atmosphere. Partitioning between root and soil respiration can shed light on linkages between variable controls of photosynthesis, autotrophic respiration, and soil respiration (including rhizomicrobial respiration carried out by heterotrophs from recent photosynthesis products) (Kuzyakov and Larionova 2005).

#### Photosynthesis and chemoautotrophic processes

Microorganisms capable of CO_2_ fixation (photoautotrophic prokaryotes and micro-eukaryotes) seem to be ubiquitous in surface soils (Cano-Díaz et al. 2020; Oliverio et al. 2020; Bay et al. 2021). The contribution of phototrophic microorganisms, i.e., direct C uptake through photosynthesis, to C fluxes in peatlands is still largely unknown. Hamard et al. (2021) estimated that these microorganisms are responsible for 10% of C uptake, which roughly equals the magnitude of projected peatland C loss due to climate warming. To the best of our knowledge, no study investigated how the structure and activity of phototropic communities are affected by rewetting to date (Table S2). These organisms can be free-living in the upper few millimeters of soil, and some are associated with *Sphagnum* mosses, where they can be highly abundant (Gilbert et al. 1998; Gilbert and Mitchell 2006; Hamard et al. 2021; Jassey et al. 2013; Tian et al. 2020). We could therefore hypothesize that the recovery of phototrophic microorganisms is dependent on restoration of vegetation, both in terms of plant (e.g., *Sphagnum* mosses) composition, and light penetration to the soil (Davies et al. 2013).

An additional potential C sink in peatlands is represented by dark, non-phototrophic CO_2_ fixation. This process, driven by chemoautotrophic microorganisms which convert inorganic carbon to organic carbon, has been shown to occur in a large range of soils, including wetlands (Nowak et al. 2015). While the occurrence of dark fixation of CO_2_ by heterotrophic microorganisms in soil is generally accepted, its importance for microbial metabolism and C balance in rewetted peatlands is unknown.

#### Heterotrophic respiration

##### Aerobic respiration

Oxygen is the most thermodynamically favorable TEA for microbial decomposition of soil organic carbon (SOC) to CO_2_. Therefore, aerobic respiration occurs at oxic compartments of the peat, i.e., above water table level, at layers with O_2_-saturation in water and in the rhizosphere. Saprotrophic fungi, actinobacteria and methanotrophs are the main aerobic decomposers in peatlands (Dedysh et al. 2006; Thormann 2006; Peltoniemi et al. 2012). Diversity and prevalence of these organisms can be an indicator of decomposition processes. For example, greater richness of saprotrophic fungi was reported under drier conditions, which may stem from both the higher prevalence of oxygen, or high fungal tolerance to drought in diverse soils (Yuste et al. 2011; de Vries et al. 2012; Barnard et al. 2013; Asemaninejad et al. 2017; Jassey et al. 2018).

Microbial C mineralization is mediated by a suite of intracellular and extracellular enzymes, with the phenol oxidase enzyme considered a key regulator (Freeman et al. 2001). Phenol oxidases degrade phenolic compounds in oxic conditions, enhancing SOC decomposition under drained conditions. In contrast, under anoxic conditions, phenolic compound accumulation limits C mineralization. However, the enzyme latch theory has been disputed as many studies show contradictory results (Laiho 2006; Turetsky et al. 2011; Carter et al. 2012; Wang et al. 2017; Urbanová and Hájek 2021). Other well-documented controls of aerobic respiration include temperature and availability of micro- and macronutrients. A less studied control that may play a significant role in CO_2_ release in rewetted peatlands is substrate quality, particularly that of C. In boreal peatlands, litter type appears to be a more important controlling factor of fungal and actinobacterial communities than water table levels (Peltoniemi et al. 2009, 2012; Strakova et al. 2011). The increased content of carboxylic, aromatic, and phenolic compounds in peat due to drainage-induced oxic conditions stimulates fungi to secrete extracellular enzymes for degradation (Peltoniemi et al. 2009).

A handful of studies have investigated aerobic respiration in rewetted peatlands, more specifically the community structure of main microbial decomposers. Fungal abundance (measured through specific membrane fatty acid quantities) was shown to increase after rewetting in the top peat layer but did not reach pristine levels (Groß-Schmölders et al. 2022). Although fungi are main decomposers in the uppermost peat layers due to their competitive advantage over bacteria, they also show higher sensitivity to changes in substrate quality, which may affect SOC decomposition in rewetted peatlands. In coastal peatlands, arbuscular mycorrhiza can be important indicator because in such habitats, plants depend more strongly on mycorrhiza as they need them to avoid salt stress (Dastogeer et al. 2020). Likewise, changes in arbuscular mycorrhiza abundances can indicate temperature change in peatlands because increase in temperature positively contributes to their growth and functions (Wang et al. 2021). Defrenne et al. (2023) demonstrated that drainage significantly changed the dominant type of mycorrhizal association: in the vicinity of ditches, it abruptly shifted from ericoid mycorrhiza to ectomycorrhiza. Most likely, changes in abundancy ratios of different mycorrhizal types in drained peatlands can indicate peat losses. In addition, bacterial to fungal ratios in the context of decomposition processes have been linked to C storage potentials (measured through ^13^C incorporation in bulk soil organic matter) in grassland soils (Malik et al. 2016), highlighting the value of combining molecular and biogeochemical techniques to understand C cycling processes and ecosystem functioning. Such an approach could be used in peatland research to assess the status of rewetted peatlands for C cycling. Investigations into aerobic respiration in rewetted peatlands have been carried out using biodiversity analyses (16S and 18S rRNA genes) and other biomarkers, such as membrane fatty acid quantities and enzyme activity assays. In an arctic peatland, metagenomics has also proven to be a useful indicator of changes in functions involved in aerobic respiration (e.g., cytochrome oxidases) across the peat profile (Lipson et al. 2013).

##### Anaerobic respiration

**Fig. 2:**
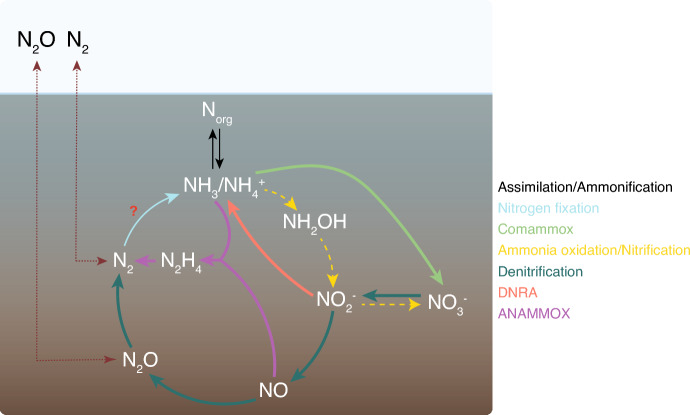
Soil processes involved in nitrous oxide (N_2_O) fluxes and expected effects of peatland rewetting. *Comammox* complete ammonia oxidation; *DNRA* dissimilatory nitrate reduction to ammonium; *ANAMMOX* anaerobic ammonium oxidation. Dashed lines correspond to pathways expected to decrease their contribution to GHG fluxes after rewetting and thick lines to increase their contribution. Dotted lines represent gas fluxes. Questionmark highlights uncertainties regarding the expected effect of rewetting due to lack of information

In peat layers where oxygen is depleted, anaerobic respiration is the main process generating CO_2_. Anaerobic microorganisms perform complex redox reactions, driving the coupling of elements, and anaerobic respiration is considered to be one of the most flexible and diverse metabolic processes. Different TEAs can be used by the resident microbial community instead of O_2_ and their order of use is broadly regulated by differences in the Gibbs free energy of the respective respiration processes and the bioavailability of TEA and electron donors (Fig. 2). The order of preference for TEA based on Gibbs free energy is NO_3_^−^/NO_2_^−^ > Mn(IV) > Fe^3+^ > sulfate (SO_4_^2−^) > organic substances. It is noticeable that the ability of organic substances to mediate redox processes was described for quinones already in the beginning of the twentieth century (Erdtman 1933). Other microbially driven pathways such as fermentation contribute to CO_2_ production in peatlands. Fermentative processes are diverse and occur via the cooperation of different functional microbial groups: primary fermenters hydrolyzing plant polymers and fermenting the monomers (such as sugars). Secondary fermenters then turn the resulting organic acids into acetate, H_2_ and CO_2_, subsequently feeding methanogenesis (see section below).

##### Nitrite (NO_2_^−^)/Nitrate (NO_3_^−^)

Canonical denitrification, i.e., the reduction of NO_2_^−^/NO_3_^−^ via nitric oxide (NO) and nitrous oxide (N_2_O) to molecular nitrogen (N_2_), contributes to SOC degradation and subsequent CO_2_ production, while also having a significant role in N_2_O production in peatlands. This process remains poorly investigated for its contribution to CO_2_ emissions from rewetted peatlands (Table S2), however denitrification is discussed in more detail in the section below in the context of N_2_O dynamics.

##### Metals

Manganese and iron are less studied TEAs in the context of peatland rewetting. A small number of studies have explored the role of manganese in the C cycle in general, one referring to Mn playing a role in C decomposition in forest ecosystems (Keiluweit et al. 2015) and another showing associations between Mn and proportions of fungal and microbial communities in an Australian peatland (Birnbaum et al. 2023). Humic and fulvic substances, abundant in peatlands, contain iron and there is increasing evidence for a role of Fe in the C cycle of peatlands both via microbial and abiotic processes. Recent literature highlights both the role of Fe reducing mineralization of SOC in peatlands upon oxia and accelerating its decomposition upon anoxia. Iron oxidation has been suggested to protect SOC in peatlands by increasing the sorption of lignin derivatives and decreasing phenolic oxidase activities (“Iron gate” theory; Wang et al. 2017). Under anoxic conditions, Fe^2+^ additions have been shown to lead to increased SOC decomposition by increasing the phenol oxidative activity most likely due to the production of the hydroxyl radical (OH), which stimulates phenol oxidase and functions as a general oxidant for organic compounds (van Bodegom et al. 2005; Halliwell and Gutteridge 2007; Wen et al. 2019). Fluctuating water levels may lead to repeated redox reactions, where Fe^2+^ is oxidized to Fe^3+^ upon oxic conditions and reduced back to Fe^2+^ as a TEA during high water level—driven anoxia. The levels of iron in peatlands can exhibit significant variability, both within individual peatlands and across different peatland locations. The cycling of iron is intricately connected to the cycling of sulfur and phosphorus. Consequently, microbial processes such as nitrification-denitrification, desulphurization, and other related metabolic processes can display considerable fluctuations in peatland environments (Dollhopf et al. 2005; Zak et al. 2021). Investigating microorganisms involved in iron reactions with typical gene-targeted approaches such as amplicon analyses remains challenging, mainly because of the lack of specific primers for iron-related functions. However, there are molecular methods available that could help alleviate these limitations (e.g., untargeted sequencing approaches).

##### Sulfate (SO_4_^2−^)

Various elements of the C cycle in peatlands can be altered by SO_4_^2−^ loading, including primary production, C mineralization and the production and export of DOC (Zak et al. 2021). SO_4_^2−^ reducing microorganisms influence C fluxes in peatlands by coupling dissimilatory SO_4_^2−^ reduction (SO_4_^2−^ to sulfide H_2_S) with heterotrophic respiration or CO_2_ fixation. Dissimilatory SO_4_^2−^ reduction is a significant contributor to SOC mineralization in peatlands (up to 36%), depending on sulfur deposition by rain or groundwater (Vile et al. 2003; Blodau et al. 2007; Deppe et al. 2010).

In comparison to other TEAs, more research has been done regarding SO_4_^2−^ and anaerobic respiration in pristine peatlands, and microbial processes involved are well characterized. To reduce SO_4_^2−^, some bacteria and archaea encode two key enzymes: the dissimilatory (bi)sulfite (*dsrAB*) and adenosine-5’-phosphosulfate reductases (*apsA*). Specific primers exist for these and dissimilatory SO_4_^2−^ reduction is relatively well studied in peatlands, generally. In comparison to other TEAs, dissimilatory SO_4_^2−^ reduction has also been explored in rewetted peatlands to some extent. Those studies have shown that O_2_ concentration and SO_4_^2−^ availability are key factors controlling the presence of SO_4_^2−^ reducers in rewetted peatlands. Furthermore, sulfate-reducing bacteria are known to tolerate a broad range of temperature and pH conditions although higher rates occur at higher temperature and neutral pH conditions (Neculita et al. 2007; Koschorreck 2008).

Higher proportions of SO_4_^2−^ reducers were detected after long-term rewetting compared to drained states (He et al. 2015; Weil et al. 2020) due to restored waterlogged and anaerobic conditions. Genes involved in SO_4_^2−^ reduction were stratified across the peat profile based on gene abundance (i.e., lower relative abundance in top layer due to presence of oxygen; Emsens et al. 2020). In addition, increased availability of SO_4_^2−^ was suggested to have led to increased CO_2_ production from a rewetted coastal peatland that received SO_4_^2−^ through inflow of brackish water (Gutekunst et al. 2022). When CO_2_ is not produced in aerobic peat decomposition during or from CH_4_ oxidation, SO_4_^2−^ input remains the strongest CO_2_ producer in such ecosystems (Knorr et al. 2008). Independent of the metabolic pathway involved, increased SO_4_^2−^ availability might alter the extent of anaerobic C mineralization, i.e., CO_2_ and CH_4_ production. Anaerobic C mineralization rates could increase relative to acetoclastic, hydrogenotrophic, and methylotrophic methanogenesis when microbes can use an electron acceptor with a higher free-energy yield such as SO_4_^2−^ (Sutton-Grier et al. 2011; Dean et al. 2018).

By coupling amplicon SIP (stable isotope probing) and 16S rRNA amplicon sequencing, Pester et al. (2010) showed that low abundance microorganisms participate in important biogeochemical cycling functions related to sulfur in peatlands. This may have been missed by typical molecular biology methods such as 16S rRNA amplicon sequencing alone.

##### General anaerobic microbial communities

Succession of redox reaction is governed by thermodynamics, but also by the distribution of TEAs which is stratified with depth as the microbial communities and associated decomposition processes are (Andersen et al. 2013). For example, in both pristine and rewetted peatlands, the anaerobic CO_2_ production rate was stratified with depth in both peatland types, being significantly higher in the surface than deeper peat layer (Urbanová and Bárta 2020).

Findings from biodiversity studies employing 16S rRNA amplicon sequencing can serve as indicators to assess the recovery of pristine-like conditions concerning dominant anaerobic microbial communities post-rewetting. A study in rewetted fens showed an increased relative abundance of anaerobic microbial groups compared to pristine peatlands (Weil et al. 2020). However, in another peatland, the anaerobic microbial community did not fully recover, likely related to a not fully restored vegetation cover and low accumulation of new peat 7–16 years after rewetting (Urbanová and Bárta 2020).

In recent years, there has been significant growth in our understanding of the processes influencing soil CO_2_ emissions and the biogeochemical and microbial factors that control them. Despite these advances, translating this knowledge into practical applications for predicting CO_2_ emissions from rewetted peatlands poses a persistent challenge. Anticipated changes in both plant community succession and microbial communities suggest a potential reduction in the radiative forcing of rewetted peatlands over time (Wen et al. 2018; Antonijević et al. 2023). Furthermore, many studies on the restoration of peatlands have documented extreme events as flooding or droughts, which may reset microbial successions (Wen et al. 2018).

### Methane

CH_4_ has taken a central role in endeavours to examine GHG fluxes in rewetted peatlands, primarily owing to its elevated radiative forcing compared to CO_2_ and its more substantial contribution to the atmospheric GHG pool than N_2_O. Peatland rewetting has been shown to increase CH_4_ emissions (Abdalla et al. 2016), with emissions factors defined by the IPCC for rewetted peatlands ranging from 41 to 216 kg CH_4_–C ha^−1^ yr^−1^ (IPCC 2014). Although water table is a major driver of CH_4_ emissions, peat properties, vegetation type, nutrient availability, climate, land-use, and restoration methods are also important factors influencing CH_4_ emissions after rewetting (Le Mer and Roger, 2001; Wilson et al. 2016; Huth et al. 2020; Tiemeyer et al. 2020; Emsens et al. 2021; Zak and McInnes 2022). Furthermore, in general, fens generate larger emissions than bogs (Abdalla et al. 2016).

CH_4_ fluxes in peatlands are maintained by a balance of taxonomically diverse aerobic methanotroph and anaerobic methanogen populations, and their respective metabolic activities. Methanogenesis is an anaerobic process in biomass decomposition and occurs where TEAs with higher thermodynamic efficiency are depleted or missing. Substrates for methanogenesis are CO_2_, hydrogen (H_2_), acetate, and methylated compounds (Fig. 1; Conrad 2020). Methanogens and their activity, commonly measured through *mcrA* (methyl coenzyme M reductase) gene abundance and expression, have consistently been shown to be significantly reduced in drained peatlands (Yrjälä et al. 2011; Urbanová et al. 2013; Urbanová and Bárta 2020). On the other hand, rewetting is expected to increase long-term CH_4_ production in peatlands because anoxia favors methanogenesis. However, diverging responses of CH_4_-cycling communities to rewetting have been observed (recently reviewed by Kitson and Bell 2020; Table S2). Effects on CH_4_ production and consumption patterns differed between peatland types (fens vs. bogs), but also within the same peatland type, where CH_4_ and related microbial community dynamics were site-specific. This is thought to be mainly due to the different types of methanogenesis prevalent in each peatland type (acetoclastic or acetate-dependent vs. hydrogenotrophic or H_2_–CO_2_-dependent methanogenesis), different controls of respective methanogenic pathways, timescale post-rewetting, and climate.

The variation in responses has been further illustrated in recently published studies (Kitson and Bell 2020). Urbanová and Bárta (2020) showed that methanogenic communities in bogs and spruce mires reached a pristine-like state 7–16 years after rewetting, while resulting CH_4_ production rates varied between the peatland types. In this study, the number of methanogens was positively correlated only with pH in rewetted spruce swamps (Urbanová and Bárta 2020), and other parameters such as water level and vegetation only indirectly affected the recovery of methanogenic activity. Rather, the degree of peat decomposition and substrate availability were critical controls of methanogenesis after rewetting, whereby low substrate availability from decomposed peat limited methanogenic activity. Thus, SOC content is thought to be an important control of CH_4_ related processes. In addition, the presence of iron oxides led to a rise in CH_4_ production in incubations of eutrophic peats upon inundation, likely due to the increased relative abundances of methanogens (de Jong et al. 2020). In coastal rewetted fens, CH_4_ production decreased after inflow of brackish water containing sulfate, which stimulates high abundance of sulfate reducers and suppressing methanogens abundance and activity (due to competition for the same substrates such as hydrogen and acetate) but not their abundance (Weil et al. 2020; Gutekunst et al. 2022). pH has also been identified as an important control of methanogenesis in peatlands, as it impacts the chemical status of substrates for methanogenesis (acetate vs. acetic acid).

CH_4_ oxidation, acting as biological methane sink in peatlands, is carried out by aerobic methane oxidizing bacteria and anaerobic methanotrophic (ANME) archaea. Aerobic methane oxidation is catalyzed by particulate and soluble methane monooxygenases (pMMO and sMMO are encoded by *pmoA* and *mmoX* genes, respectively), which require molecular oxygen as terminal electron acceptor (Ross and Rosenzweig 2017). Active aerobic methanotrophs are therefore found along the oxic-anoxic boundary in the upper peat layers, at the vascular plant root/peat interface and associated with *Sphagnum* mosses (Raghoebarsing et al. 2005). Anaerobic methanogens use the reverse reaction of the canonical final step in the methanogenesis pathway (Krüger et al. 2003; Hallam et al. 2004).

Kitson and Bell (2020) reviewed responses of methanotrophs to rewetting and similarly to methanogenic communities, findings varied across studies. Lower abundances of methanotrophs were observed in rewetted compared to pristine fens and bogs. In addition, the recovery of methanotrophic populations was slow compared to the one of methanogens (measured through particulate methane monooxygenase or *pmoA* gene abundance; Putkinen et al. 2018; Wen et al. 2018; Emsens et al. 2020). This could be explained by disturbances to the oxic–anoxic boundary zones following inundation and whether revegetation is part of the restoration efforts. In fact, the recovery of type II methanotrophs was shown to be strongly linked to *Sphagnum* abundance (Putkinen et al. 2018) and plant succession (Urbanová and Bárta 2020). Since the review by Kitson and Bell (2020), recent research reported that in rewetted coastal fens, methanotroph abundances reached pre-drought (i.e., near natural) levels after inflow of brackish water, while low but unaffected levels of methane oxidation were observed (Gutekunst et al. 2022). In addition, anaerobic methane oxidation (from ANME archaea) in NO_2_^−^/NO_3_^−^ rich boundary layers was suggested to substantially lower methane release in wet peatlands (Zhu et al. 2012; Miller et al. 2019) and rewetted organic soils (Legierse et al. 2023).

While the focus on CH_4_ is dominant and processes related to this GHG are well characterized on a genetic and molecular level compared to other processes, uncertainties remain on CH_4_ emissions from rewetted peatlands. Findings altogether illustrate the variability in responses of CH_4_-cycling communities and functions post-rewetting, which appear to be governed by a variety of factors and are peatland specific (Weil et al. 2023). Relationships between peat depth, decomposition state in relation to availability of substrates for CH_4_ related microbial processes still need to be further investigated.

### Nitrous oxide

The effects of restoring peatlands on N_2_O emissions have yet to be fully understood (expected outcomes of rewetting are shown in Fig. 2). While the IPCC’s default methodology assumes that restoring peatlands will reduce emissions to nearly zero (IPCC 2014), a recent meta-analysis of studies showed that soil N_2_O emissions from rewetted European peatlands can range from − 1.08 to 5.27 kg N_2_O–N ha^−1^ yr^−1^ (Lin et al. 2022). In general, restoring peatlands can be considered an effective method for lowering N_2_O emissions from drained nutrient-rich peatlands (Lin et al. 2022; Liu et al. 2020; Minkkinen et al. 2020). However, the effects of restoration may vary depending on the type and degradation state of the peatland, land-use history of drained peatland prior to rewetting, and the time since rewetting, as well as the restoration method used. In some cases, rewetted soil may still show high N_2_O release rates, especially if fertilized (Kandel et al. 2019, Liu et al. 2019).

The nitrogen cycle is largely performed by microorganisms, and many microbial processes are known to be sources of N_2_O (Fig. 2; Kuypers et al. 2018). In water saturated systems, including peatlands, denitrification usually represents the main source of N_2_O, favored by O_2_ depletion from high water table levels and/or high organic C content (Conrad 1996; Pihlatie et al. 2004; Palmer et al. 2010). Like in pristine peatlands, denitrification is also the main source for N_2_O after rewetting as demonstrated in a short-term flooding experiment, due to restoration of anoxic conditions (Masta et al. 2022, 2023). However, depending on the peatland type a large share of N_2_O may also have originated from nitrifier denitrification as demonstrated recently for a rewetted coastal peatland (Behrendt and Wrage-Mönnig 2023). The increase in N_2_O emissions was correlated to higher gene copy numbers of denitrification genes (*nirK*, *nirS* and *nosZ*) with evidence of incomplete denitrification occurring. These results are in contrast with results of a drought/flooding experiment by Palmer et al. (2016). Authors showed that in situ N_2_O emissions were low and fluxes stable during flooding due to higher rates of complete denitrification, which is consistent with other work in both rewetted bogs (Tauchnitz et al. 2015) and freshwater marsh (Yang et al. 2013). This divergence highlights the variability of N-cycling response to peatland rewetting, partially likely due to initial differences in peat nutrient conditions, including external inputs of N by deposition or with freshwater. It has been shown that N_2_O production caused by denitrification is mainly controlled by the availability of NO_3_^−^ in relation to water table levels (Tauchnitz et al. 2015), and that NO_3_^−^ loading causes increased N_2_O emissions in rewetted peatlands (Russow et al. 2013), as well as pristine peatlands (Palmer and Horn 2015), and organic soils (Pärn et al. 2018).

To the best of our knowledge, the studies by Masta et al. (2022 and 2023) represent the only ones to date linking N_2_O fluxes with ammonia oxidation (by using nitrification gene *amoA* abundances as indicator) in rewetted peatlands (Table S2). There, ammonia oxidation was estimated to be a source for N_2_O, secondary to denitrification. Globally, the contribution of nitrifying microorganisms to N_2_O fluxes in peatlands is thought to be substantial and underestimated (Siljanen et al. 2019; Bahram et al. 2022). Ammonia oxidizing microorganisms are believed to hold a pivotal role in N_2_O emissions because producing both N_2_O indirectly (from abiotic transformation of hydroxylamine) and NO_3_^−^ (substrate for denitrification and subsequently more N_2_O produced), however their contribution to N_2_O fluxes in pristine as well as rewetted peatlands is still understudied. In addition, complete oxidation of ammonium to NO_3_^−^ (comammox; Daims et al. 2015; van Kessel et al. 2015) still needs to be investigated to assess their contribution to N_2_O fluxes and their role in many ecosystems (in ‘t Zandt et al. 2018), not to mention natural or peatlands under restoration. The importance of these processes as a source for N_2_O is anticipated to diminish in the initial years. This decline is attributed to the fact that the ammonium pool, resulting from prior mineralization processes under drained conditions, does not undergo replenishment in rewetted conditions. Furthermore, nitrate is expected to disappear at an accelerated rate, typically within a few weeks to months, as outlined by Zak et al. (2010).

Higher N_2_O emissions after rewetting were also correlated with increased DNRA (dissimilatory nitrate reduction to ammonium; Espenberg et al. 2018; Jahangir et al. 2020; Masta et al. 2022). *nrfA* (cytochrome c nitrite reductase) gene copies were positively correlated with N_2_O emissions both in pristine and rewetted peatlands, further highlighting the contribution of this process to N_2_O fluxes. While typically a process considered conserving N in the ecosystem, DNRA can also represent a source of N_2_O as a byproduct of the reduction of NO_3_^−^ to ammonia. This process is favored in competition with denitrifiers when NO_3_^−^ concentrations are low, the latter being most likely related to the inherent peat chemical composition prior to rewetting, as also discussed above for denitrification.

Complete heterotrophic denitrification is the main biological process known as a sink for N_2_O, through the last step of denitrification performed by the nitrous oxide reductase Nos (encoded by *nosZ* clade I and *nosZ* clade II genes; Hallin et al. 2018). While controls of this process are critical to consider for mitigating N_2_O emissions from peatlands, the effect of rewetting on N_2_O-related genes and enzymes remains poorly studied. The absolute requirement of Nos for copper for activity, as well as the absence of any parallel pathways that can reduce N_2_O, account for the critical role of this element in the success of the final step of denitrification (Richardson et al. 2009). Additionally, pH has been shown to represent an important factor for *nosZ* enzyme maturation (Bakken et al. 2012), which is essential to consider as water pH can be impacted by rewetting (Lundin et al. 2017). In a study by Masta et al. (2022), flooding peat led to a concurrent increase in *nosZ* activity and N_2_O emissions. In this study, the ratio of *nosZ* to *nirS* and *nirK* gene proportions indicated incomplete denitrification, possibly explaining high N_2_O emissions. The in situ flooding-drying experiments in a drained peatland forest (Masta et al. 2023) revealed that denitrification dominated the small emission of N_2_O under flooded conditions, possibly reduced by complete denitrification (increased *nosZ* genes abundance), whereas drained peat emitted significantly more N_2_O. In the last case, ammonia oxidation was the main N_2_O source which was indicated by elevated abundance of bacterial, archaeal and comammox *amoA* genes. This has also been suggested in restored agricultural wetlands (Kasak et al. 2021). While nosZI-type denitrifiers play an important role in controlling N_2_O and N_2_ gas fluxes in both natural and rewetted peatlands, it should be noted that slightly more than half of clade II nosZ organisms are apparently non-denitrifying N_2_O reducers and therefore have the potential to be a sink without contributing to N_2_O release (Hallin et al. 2018; Espenberg et al. 2018). Recently, the novel species *Flavobacterium azooxidireducens* sp. nov. was isolated from a *Phragmites* litter decomposition experiment which was able to consume significant amounts of N_2_O under anaerobic conditions (Behrendt et al. 2022). The Nos enzyme is also used in n-damo pathway (nitrite dependent anaerobic methane oxidation), where NO_2_^–^/ NO_3_^–^ is reduced to N_2_ and CH_4_ is anaerobically oxidized to CO_2_ (Raghoebarsing et al. 2006). The effect of rewetting on microorganisms possessing n-damo capacities has not been investigated in rewetted peatlands to date.

The vast majority of studies investigating N_2_O related processes in rewetted peatlands have used gene-targeted approaches, mainly qPCR, focusing on known key N-cycling functions. However, soil microbial communities are generally highly diverse and contain massive unknown taxonomic and functional diversity (see paragraph below). There are few studies about natural and restored peatlands synthesizing different methods like metagenomics, qPCR, N_2_O and N_2_ emissions (Espenberg et al. 2018; Bahram et al. 2022) or qPCR, isotopes and N_2_O emissions (Masta et al. 2022, 2023). The use of untargeted molecular approaches such as metagenomics and -transcriptomics would help (i) explore the unknown microbial diversity, (ii) detangle the complex networks of processes behind GHG emissions in peatlands. Recent examples of new genes/processes discovered using these molecular techniques and involved in biogeochemical cycles include the identification of comammox (Daims et al. 2015) and *nosZ* clade II (Sanford et al. 2012). Moreover, in the study by Palmer et al. (2016), rewetting-driven fluctuations in water table/oxygen content resulted in impacts on microbial activity (of denitrifiers) rather than community composition. The metabolic flexibility of most denitrifiers, together with high functional redundancy in soil microbial communities highlight the need to look at key gene expression rather than taxonomic biodiversity to better understand N_2_O-related processes in rewetted peatlands (see section below on molecular methods).

While the rewetting of drained peatlands can stimulate microbial nitrogen cycling processes and lead to associated N2O production, as mentioned earlier, a swift decline is anticipated once ammonium and nitrate are depleted and not replenished by contaminated ground or surface water. This needs to be considered when developing viable management options to reduce N_2_O emissions from drained peatlands. However, the magnitude of N-cycling and changes in the N_2_O production to consumption balance (i.e., the N_2_O product ratio of denitrification) is strongly affected by peat nutrient conditions, availability of NO_3_^−^ and other electron acceptors (e.g., Fe, SO_4_^2−^) and water table depth and its fluctuations (de Jong et al. 2020). Many environmental factors that govern N_2_O fluxes at a large scale in pristine peatlands (e.g., soil C/N ratio, temperature, pH, peat type, climate zones, and vegetation cover; Martikainen et al. 1993; Repo et al. 2009; Shi et al. 2021; Yao et al. 2022) haven’t been addressed in rewetted peatlands. Site dependences of microbial N cycling responses most likely explain why some studies observed a net N_2_O uptake by rewetted peatlands (Berendt et al. 2023, Ye and Horwath 2016). Still, the underlying controls over N_2_O consumption and the capacity of rewetted peat soils to act as potential sink for atmospheric N_2_O are poorly understood.

## Potentials of molecular biology tools in peatland research

**Fig. 3:**
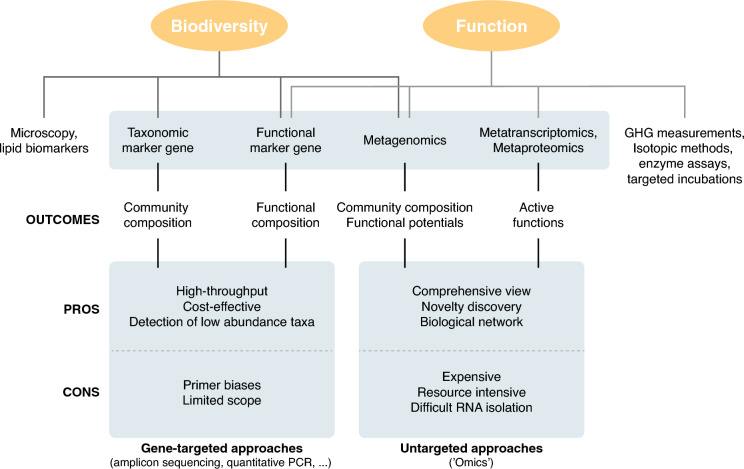
Overview of molecular biology methods and their respective “Pros and Cons” in investigating microbial diversity and biogeochemical processes. Emphasis is placed on exploring microbial functions linked to greenhouse gas (GHG) emissions in rewetted peatlands. (*PCR* polymerase chain reation)

Measuring GHG fluxes and their controls on the field and at large scale involves tedious and expensive work and GHG are mainly end- or by-products of biotic processes. Due to the complex network of GHG production and consumption processes, it is challenging to trace GHG emissions to discrete processes. Alternatives for assessing restoration success, or prior to restoration to predict its impact on microbial functions and GHG dynamics, could include a broader use of molecular methods and the development of a wider suite of microbial molecular biomarkers (Fig. 3). This is especially relevant because of the variation in peatland response to rewetting in terms of GHG emissions highlighted in this review.

Many plant biomarkers have been established based on detection of compounds via GC/MS (e.g., lignin, polysaccharides, N compounds, etc.) to assess peat chemical composition and plant composition in natural and drained peatlands (reviewed in bogs by Klein et al. 2022). Fewer biomarkers for microbial transformations exist. Studies focusing on drained or rewetted bogs and fens employed methods such as PLFA (phospholipid-derived fatty acids) analyses for estimation of total biomass and broad changes in community composition (Xu et al. 2021; Groß-Schmölders et al. 2021, 2022). However, this type of analysis only targets specific taxonomic groups of organisms, or broad groups (e.g., all bacteria). Additionally, enzyme activity assays have been used to assess microbial activity in peat, but they have to date mostly been applied to near-natural or drained peatlands (Xu et al. 2021; Xue et al. 2021). Molecular tools such as high-throughput sequencing can contribute greatly to understanding peat microbial taxonomic and functional diversity. Whether utilizing DNA- or RNA-based methods, whether employing targeted or untargeted approaches, integrating these tools into peatland research holds promise for evaluating restoration status and ecosystem functioning. These methodologies have demonstrated efficiency in investigating ecosystem functioning in diverse environments, underscoring their applicability and value in peatland studies.

Commonly used 16S rRNA gene surveys focus on characterizing taxonomy and community structure rather than function. However, gene abundance and activity better represent peatland processes than species presence/absence for several reasons: (i) DNA-based analyses are not representative of active microbial communities due to the presence of legacy DNA. DNA from dormant or dead cells leads to biases as cell mortality/dormancy and subsequently legacy DNA might increase when physicochemical conditions change drastically and/or reoccurring environmental stresses (e.g., drought/rewetting cycles). (ii) High functional redundancy exists in soil microbial communities (Chen et al. 2022). Moreover, gene-targeted methods currently used in peatland research limit investigations exploring the so-called ‘microbial dark matter’ (i.e., the enormous diversity of yet-uncultivated microorganisms), that most likely play important roles in biogeochemical cycles. There is still a huge pool of unknown taxonomic diversity and microbial functions in soils, including peat, that remains to be assessed and that represents potential reservoirs of functions impacting nutrient recycling and GHG fluxes directly (catalytic enzymes) or indirectly (gene regulators).

While RNA-based analyses have become popular to generate information of active members and functions in microbial communities and thus describe ecosystem processes, they are chiefly lacking in peatland research. This is mainly due to the difficulty of recovering RNA from peat, because of high humic acid content inhibiting traditional nucleic acid extraction protocols and downstream analyses such as PCR amplification. No metatranscriptomic studies assessing changes in active functions after rewetting and compare to pristine peatlands were found in our literature search, but some have been carried out in natural peatlands (Lin et al. 2014; Hausmann et al. 2019). Additionally, other metatranscriptomic analyses have focused so far on *Sphagnum* associated microbiomes (Ivanova et al. 2018; Stough et al. 2018; Dedysh and Ivanova 2019; Carrell et al. 2022a, b; Kolton et al. 2022), arctic peatlands (Tveit et al. 2013, 2014, 2015; Belova et al. 2018; Dedysh and Ivanova 2019; Ziegelhofer and Kujala 2021; Bender et al. 2021) and specific microbial groups such as protists (Geisen et al. 2015) and Planctomycetes (Ravin et al. 2018; Ivanova et al. 2018; Dedysh and Ivanova 2019). Additionally, in cases where peat chemical composition cannot be measured, metatranscriptomics could prove helpful as the function of active genes can provide information to some extent regarding what substrate is being used (with the caveat that information in databases used to functionally annotate genes are limited).

## Conclusion and future directions

Up to now, precisely predicting trajectories of changes in the net C and GHG balance of rewetted peatlands remains difficult. That is largely due missing tools for rapid assessments of changes in microbial processes and communities in response to water status changes. The utilisation of rapidly advancing technologies, such as high-throughput sequencing, is poised to enhance our comprehension of soil microbial diversity, as well as the physiological abilities and roles of individual taxa in rewetted peatlands. Eventually, this information on microbial ecology can be used for narrowing down future outcomes of a particular rewetted peatland in terms of GHG dynamics.

To achieve this vision, further research is needed on:

- Changes in microbial community structure and function across peat depth, vegetation types and degree of degradation, in combination with multi-dimensional (spatial and temporal) assessments of GHG production, consumption and emission dynamics; more emphasis should be placed on the indicator values of microbial and fungal communities to detect changes in environmental factors in peatlands.
- Processes starting from substrate concentrations towards active microbial functions, up to GHG dynamics in rewetted peatlands using e.g., isotope tracing approaches.
- Combining different methods like qPCR, metagenomics, metatranscriptomics, isotopes and GHG analyses to study and validate microbial functions and community.

A more harmonized approach, linking similar molecular biology methods with biogeochemistry on rewetted peatlands with variable characteristics, including time before/after restoration and measurement methods and intensity, would facilitate calibrating this rapidly evolving research tool as a bioindicator for restoration outcomes.

### Supplementary Information

Below is the link to the electronic supplementary material.

Supplementary material 1 (XLSX 85.1 kb)

Supplementary material 2 (PDF 110.9 kb)

## Data Availability

There are no original data associated with this paper.
