# Supplementary material for: Unraveling microbial processes involved in carbon and nitrogen cycling and greenhouse gas emissions in rewetted peatlands by molecular biology
Source: Biogeochemistry. 2024 Mar 16;167(4):609–29. doi: 10.1007/s10533-024-01122-6 (PMC11068585; doi:10.1007/s10533-024-01122-6)
Supplement: Supplementary file 2 — Supplementary material 2 (PDF 110.9 kb) [file 10533_2024_1122_MOESM2_ESM.pdf]

References for Table S1

Antonijević D, Hoffmann M, Prochnow A, et al (2023) The unexpected long period of elevated CH_4 emissions from an inundated fen meadow ended only with the occurrence of cattail (*Typha latifolia*). Glob Chang Biol 00:1–14. https://doi.org/10.1111/gcb.16713

Cabezas A, Pallasch M, Schoenfelder I, Gelbrecht J, Zak D (2014). Carbon, nitrogen, and phosphorus accumulation in novel ecosystems: Shallow lakes in degraded fen areas. Ecol Eng 66:63–71. https://doi.org/10.1016/j.ecoleng.2013.10.037

Eggelsmann R, Heathwaite AL, Grosse-Braukmann G, et al (1993) Physical processes and properties of mires. In: Heathwaite AL, Gottlich K (eds) Mires, process, exploration and conservation. John Wiley, Chichester, pp 171-262

Freeman C, Ostle N, Kang H (2001) An enzymic 'latch' on a global carbon store - shortage of oxygen locks up carbon in peatlands by restraining a single enzyme. Nature 409:149. https://doi.org/10.1038/35051650

Gaffnet PP, Hancock MH, Taggart MA, Andersen R (2018) Measuring restoration progress using pore- and surface-water chemistry across a chronosequence of formerly afforested blanket bogs. J Environ Manag 219:239–251. https://doi.org/10.1016/j.jenvman.2018.04.106

Haapalehto TO, Vasander H, Jauhiainen S, Tahvanainen T, Kotiaho JS (2011) The effects of peatland restoration on water-table depth, elemental concentrations, and vegetation: 10 years of changes. Restor Ecol 19:587–598. https://doi.org/10.1111/j.1526-100X.2010.00704.x

Hahn-Schöfl M, Zak D, Minke M, et al (2011) Organic sediment formed during inundation of a degraded fen grassland emits large fluxes of CH_4 and CO_2. Biogeosciences 8:1539–1550. https://doi.org/10.5194/bg-8-1539-2011

Holden J, Chapman PJ, Labadz JC (2004) Artificial drainage of peatlands: hydrological and hydrochemical process and wetland restoration. Prog Phys Geogr 28:95–123. https://doi.org/10.1191/0309133304pp403ra

Holden J, Smart RP, Dinsmore KJ, et al (2012) Natural pipes in blanket peatlands: Major point sources for the release of carbon to the aquatic system. Glob Chang Biol 18:3568–3580. https://doi.org/10.1111/gcb.12004

Janssen JAM, Rodwell JS, García Criado M, et al (2016) European Red List of Habitats: Part 2. Terrestrial and Freshwater Habitats. Publication Office of the European Union, Luxembourg

Kreyling J, Tanneberger F, Jansen F et al (2021) Rewetting does not return drained fen peatlands to their old selves. Nature Commun 12:5693. https://doi.org/10.1038/s41467-021-25619-y

Lamentowicz M, Mueller M, Gałka M, et al (2015) Reconstructing human impact on peatland development during the past 200 years in CE Europe through biotic proxies and X-ray tomography. Quat Int 357:282–294. https://doi.org/10.1016/j.quaint.2014.07.045

Lamers LPM, Roozendaal SME, Roelofs JGM (1998) Acidification of freshwater wetlands: combined effects of non-airborne sulphur pollution and desiccation. Wat Air And Soil Poll 105:95–106. https://doi.org/10.1023/A:1005083526455

Leifeld J, Klein K, Wüst-Galley C (2020) Soil organic matter stoichiometry as indicator for peatland degradation. Sci Rep 10:7634. https://doi.org/10.1038/s41598-020-64275-y

Liu H, Zak D, Rezanezhad F, Lennartz B (2019) Soil degradation determines release of

nitrous oxide and dissolved organic carbon from peatlands. Environ Res Lett 14:094009. https://doi.org/10.1088/1748-9326/ab3947

Menberu MW, Tahvanainen T, Marttila H, et al (2016) Water-table dependent hydrological changes following peatland forestry drainage and restoration: Analysis of restoration success, Water Resour Res 52:3742–3760. https://doi.org/10.1002/2015WR018578

Menberu MW, Marttila H,Tahvanainen T, et al (2017) Changes in pore water quality after peatland restoration: Assessment of a large-scale, replicated Before-After-Control-Impact study in Finland. Water Resour Res 53:8327–8343. https://doi.org/10.1002/2017WR020630

Pärn J, Verhoeven JTA, Butterbach-Bahl K, et al (2018) Nitrogen-rich organic soils under warm well-drained conditions are global nitrous oxide emission hotspots. Nat Commun 9:1135. https://doi.org/10.1038/s41467-018-03540-1

Paavilainen E, Päivänen J (1995) Peatland forestry: Ecology and principles. Springer-Verlag, Berlin Heidelberg

Rydin H, Jeglum J (2013) The biology of peatlands, 2nd edn. Oxford University Press, UK

Tarvainen O, Laine AM, Peltonen M, Tolvanen A (2013) Mineralization and decomposition rates in restored pine fens. Restor Ecol 21:592–599. https://doi.org/10.1111/j.1526-100X.2012.00930.x

Tiemeyer B, Freibauer A, Borraz EA, et al (2020) A new methodology for organic soils in national greenhouse gas inventories: Data synthesis, derivation and application. Ecol Indic 109:105838. https://doi.org/10.1016/j.ecolind.2019.105838

Tolvanen A, Tarvainen O, Laine AM (2020) Soil and water nutrients in stem-only and whole-tree harvest treatments in restored boreal peatlands. Restor Ecol 28:1357–1364. https://doi.org/10.1111/rec.13261

Verhoeven JT, Liefveld WM (1997) The ecological significance of organochemical compounds in *Sphagnum*. Acta Bot Neerl 46:117–130. https://doi.org/10.1111/plb.1997.46.2.117

Walton CR, Zak D, Audet J, et al (2020) Wetland buffer zones for nitrogen and phosphorus retention: impacts of soil type, hydrology and vegetation. Sci Total Envir 727:138709. https://doi.org/10.1016/j.scitotenv.2020.138709

Zak D, Gelbrecht J (2007) The mobilisation of phosphorus, organic carbon and ammonium in the initial stage of fen rewetting (a case study from NE Germany). Biogeochemistry 85:141–151. https://doi.org/10.1007/s10533-007-9122-2

Zak D, Gelbrecht J, Wagner C, Steinberg CEW (2008) Evaluation of phosphorus mobilization potential in rewetted fens by an improved sequential chemical extraction procedure. Eur J Soil Sci 59:1191–1201. https://doi.org/10.1111/j.1365-2389.2008.01081.x

Zak D, Wagner C, Payer B, Augustin J, Gelbrecht J (2010) Phosphorus mobilization in rewetted fens: The effect of altered peat properties and implications for their restoration. Ecol Appl 20:1336–1349. https://doi.org/10.1890/08-2053.1

Zak D, Goldhammer T, Cabezas A, et al (2018) Top soil removal reduces water pollution from phosphorus and dissolved organic matter and lowers methane emissions from rewetted peatlands. J Appl Ecol 55:311–320. https://doi.org/10.1111/1365-2664.12931

Zak D, Roth C, Unger V, et al (2019) Unraveling the importance of polyphenols for microbial carbon mineralization in rewetted riparian peatlands. Front Environ Sci 7. https://doi.org/10.3389/fenvs.2019.00147

Zak D, McInnes RJ (2022) A call for refining the peatland restoration strategy in Europe. J Appl Ecol 59:2698–2704. https://doi.org/10.1111/1365-2664.14261
